# Expression profiling of genes regulated by Fra-1/AP-1 transcription factor during bleomycin-induced pulmonary fibrosis

**DOI:** 10.1186/1471-2164-14-381

**Published:** 2013-06-07

**Authors:** Subbiah Rajasekaran, Narsa M Reddy, Wei Zhang, Sekhar P Reddy

**Affiliations:** 1 Division of Developmental Biology and Basic Research, Department of Pediatrics, University of Illinois at Chicago, 830 S. Wood Street, Chicago, IL 60612, USA

## Abstract

**Background:**

The Fra-1/AP-1 transcription factor regulates the expression of genes controlling various processes including migration, invasion, and survival as well as extracellular remodeling. We recently demonstrated that loss of *Fra-1* leads to exacerbated bleomycin-induced pulmonary fibrosis, accompanied by enhanced expression of various inflammatory and fibrotic genes. To better understand the molecular mechanisms by which Fra-1 confers protection during bleomycin-induced lung injury, genome-wide mRNA expression profiling was performed.

**Results:**

We found that Fra-1 regulates gene expression programs that include: 1) several cytokines and chemokines involved in inflammation, 2) several genes involved in the extracellular remodeling and cell adhesion, and 3) several genes involved in programmed cell death.

**Conclusion:**

Loss of Fra-1 leads to the enhanced expression of genes regulating inflammation and immune responses and decreased the expression of genes involved in apoptosis, suggesting that this transcription factor distinctly modulates early pro-fibrotic cellular responses.

## Background

Pulmonary fibrosis is a chronic, progressive, and usually untreatable group of chronic disorders and appears to be regulated by complex cellular processes [1]. In animal models, a single intratracheal administration of bleomycin induces an inflammatory response that is characterized by leukocyte infiltration, apoptosis, fibroblast proliferation, matrix metalloproteinase (MMP)/tissue inhibitor of metalloproteinase (TIMP) imbalance, and an increase in interstitial collagen content [2,3] that can culminate in the development of pulmonary lesions similar to those observed in human interstitial pulmonary fibrosis (IPF) [4]. However, the exact mechanisms underlying pulmonary fibrosis remain unclear.

AP-1 is a dimeric transcription factor, mainly comprised of the Jun (c-Jun, Jun-B, Jun-D), Fos (c-Fos, Fos-B, Fra-1, Fra-2), and ATF (ATF1-4) families of b-ZIP transcription factors. AP-1 binds to the TPA response element (TRE, also known as the AP-1 site) and regulates target gene expression in response to various pro-oxidants and toxicants. These gene products mediate (mitigate or promote) oxidative stress and inflammatory responses, as well as cell growth and tumorigenesis [5]. The promoters of many inflammatory response genes, especially those encoding cytokines and chemokines, have functional AP-1 binding sites [6]. Fra-1 regulates gene expression involved in various processes such as cell growth and cell death and regulates the expression of genes controlling tissue/cell remodeling, such as MMP-1, MMP-2, and MMP-9, mainly at the transcriptional level [7-11]. We have recently shown that Fra-1-deficient (*Fra-1*^∆/∆^) mice are more susceptible than wild-type (*Fra-1*^*+/+*^) mice to bleomycin-induced fibrosis [12], suggesting that this transcription factor is involved in the regulation of complex genetic networks to maintain cellular homeostasis during bleomycin-induced lung inflammation, injury, and repair processes. Based on these results, we hypothesized that accelerated inflammation and fibrosis observed in *Fra-1*^∆/∆^ mice are caused by enhanced inflammatory and fibrotic gene expression. To test this hypothesis and to better understand the mechanisms by which the Fra-1 transcription factor confers pulmonary protection, we have performed microarray analysis to examine the changes in gene expression in the lungs of *Fra-1*^∆/∆^ mice after treatment with bleomycin. In the present study, we have evaluated changes in early inflammatory and pro-fibrotic gene expression after 5 days of bleomycin treatment. Our mRNA expression profiling demonstrated increased expression of genes involved in inflammation and immune responses and decreased levels of apoptotic genes in *Fra-1*^∆/∆^ mice, suggesting that the Fra-1 transcription factor dampens the development of late fibrotic injury by modulating the early pro-fibrotic responses.

## Results and discussion

### Genes that encode cytokines, chemokines, and their receptors

The set of genes that was differentially expressed between PBS-treated *Fra-1*^*+/+*^ and *Fra-1*^∆/∆^ mice was studied in order to identify those genes for which a genotypic difference in expression exists. We found that the loss of Fra-1 led to an up-regulation of chemokine (C-X-C motif) ligand 13 (*Cxcl13,* 1.7-fold) and interleukin 1 alpha (*Il1a,* 1.7-fold) expression. Similarly, we found a down-regulation of chemokine (C-C motif) ligand 5 (*Ccl5,* -1.6-fold) and chemokine (C-X-C motif) ligand 9 (*Cxcl9,* -2.7-fold) in *Fra-1*^∆/∆^ mice when compared to *Fra-1*^*+/+*^ mice (Table 1). Next, we compared the differentially up-regulated genes between bleomycin-treated *Fra-1*^∆/∆^ and *Fra-1*^*+/+*^ mice (Table 2). The genes that showed a -fold change of ≥1.7 were selected for analysis. Interestingly, our data suggested that the lack of Fra-1 leads to up-regulation of cytokines and chemokines in response to bleomycin, including interleukin-1 alpha (*Il1a,* 1.87-fold), interleukin-2 receptor alpha chain (*Il2ra*, 2.15-fold), interleukin 2 receptor, beta chain (*Il2rb,* 1.69-fold), interleukin 6 (*Il6,* 2.23-fold), chemokine (C-C motif) ligand 8 (*Ccl8,* 2.27-fold), and C-X-C motif chemokine 10 (*Cxcl10,* 2.54-fold), whereas *Fra-1*^*+/+*^ mice showed an up-regulation of interleukin 1 receptor, type II (*Il1r2,* 2.27-fold). These results suggest that Fra-1 signaling controls the expression of some of the genes that are involved in fibrosis. For instance, expression of interleukin 6, a cytokine that promotes greater inflammation and fibrosis [13], was significantly higher in bleomycin-treated *Fra-1*^∆/∆^ mice (2.23-fold) than in *Fra-1*^*+/+*^ mice (Table 2). Also, we noted that *Fra-1*^*+/+*^ mice showed an increased expression of *Il1r2* (*Il1r2,* 2.27-fold) in response to bleomycin as compared to *Fra-1*^∆/∆^ mice. Interleukin 1 (*Il1*), a principal pro-inflammatory cytokine that includes two ligands (*Il1α* and *Il1β*) and two cell surface receptors namely *Il1r1* and *Il1r2*. Several reports suggest that binding of *Il1* to *Il1r1* ultimately leads to the activation of several genes, including those encoding cyclooxygenase, nitricoxide synthase, cell adhesion molecules and cytokines and chemokines [14,15]. More importantly, in mouse models, exogenous administration of recombinant *Il1β* induced high degree of bleomycin-induced fibrosis, and specific blockade of *Il1r1* markedly reduced bleomycin-induced inflammation [16,17]. Due to the lack of a cytoplasmic-signaling domain for *Il1r2,* this receptor mainly acts as a decoy receptor to prevent *Il1-*mediated biological responses [18]. Many anti-inflammatory mediators enhance the expression and release of *Il1r2* to induce anti-Il1 pathway [19-21]. The increased *Il1r2* expression in *Fra-1*^*+/+*^ but not in *Fra-1*^∆/∆^ mice suggests that Fra-1 controls bleomycin-induced inflammation by augmenting the expression of anti-inflammatory genes. In the bleomycin-induced fibrosis model, extensive neovascularization has often been observed to follow the airways and sites of injury [22]. Presence/absence of ERL motif in CXC chemokines dictates their angiogenic property [23]. The amount of Cxcl10 chemokine in the lungs has been directly correlated with the degree of fibrosis [24]. Administration of Cxcl10 to bleomycin-treated mice attenuates pulmonary fibrosis in part due to reduced angiogenesis [25]. However, we found that bleomycin-induced *Fra-1*^∆/∆^ mice showed a 2.54-fold increase in *Cxcl10* when compared to *Fra-1*^*+/+*^ mice (Table 2).

**Table 1:** **Differentially expressed genes in lung tissues of *Fra-1***^∆/∆^**and *Fra-1***^***+/+ ***^**mice**

**Affymetrix ID**	**Symbol**	**Gene title**	**KOC vs. WTC (fold change)**
Cytokine and chemokines			
10487588	Il1a	Interleukin 1 alpha	1.7
10523359	Cxcl13	Chemokine (C-X-C motif) ligand 13	1.7
10389207	Ccl5	Chemokine (C-C motif) ligand 5	−1.6
10531407	Cxcl9	Chemokine (C-X-C motif) ligand 9	−2.7
Inflammation			
10545569	Reg3g	Regenerating islet-derived 3 gamma	7
10402390	Serpina1b	Serine preptidase inhibitor, clade A, member 1B	4.61
10535559	Baiap2l1	BAI1-associated protein 2-like 1	3.9
10349648	Ctse	Cathepsin E	3.61
10402409	Serpina1e	Serine peptidase inhibitor, clade A, member 1E	2.47
10563597	Saa3	Serum amyloid A 3	1.82
10362138	Vnn1	Vanin 1	1.72
10398117	Bdkrb2	Bradykinin receptor B2	1.69
10481627	Lcn2	Lipocalin 2	1.4
10372652	Lyz1	Lysozyme 1	1.31
10444824	H2-Q6	Histocompatibility 2, Q region locus 6	−1.66
10497356	Sirpb1a	Signal-regulatory protein beta 1A	−1.72
10574149	Nlrc5	NLR family, CARD domain containing 5	−1.78
10398069	Serpina3m	Serine peptidase inhibitor, clade A, member 3 M	−2.32
Cell adhesion molecule			
10395553	Nrcam	Neuron-glia-CAM-related cell adhesion molecule	1.95
10523717	Spp1	Secreted phosphoprotein 1	−1.81
Transcription			
10521537	Cytl1	Cytokine-like 1	2.02
10598409	Tcfe3	Transcription factor E3	1.78
10583312	Taf1d	TATA box binding protein (Tbp)-associated factor, RNA polymerase I, D	−1.63
10399725	Sox11	SRY-box containing gene 11	−1.96

**Table 2:** **Differentially expressed genes induced by bleomycin in lung tissues of *Fra-1***^***+/+ ***^**and *Fra-1***^∆/∆^**mice**

**Affymetrix ID**	**Symbol**	**Gene title**	**KOT vs. WTT (fold change)**
Cytokine, chemokines, and their receptors			
10531415	Cxcl10	C-X-C motif chemokine 10	2.54
10379535	Ccl8	Chemokine (C-C motif) ligand 8	2.27
10520452	Il6	Interleukin 6	2.23
10469278	Il2ra	Interleukin-2 receptor alpha chain	2.15
10487588	Il1a	Interleukin-1 alpha	1.87
10430344	Il2rb	Interleukin 2 receptor, beta chain	1.69
10523128	Ppbp	Chemokine (C-X-C motif) ligand 7	–2.17
10345752	Il1r2	Interleukin 1 receptor, type II	−2.27
Inflammation			
10531126	Igj	Immunoglobulin joining chain	17.25
10496555	Gbp1	Guanylate binding protein 1	7.88
10403069	Igh-6	Immunoglobulin heavy constant mu	7.43
10545569	Reg3g	Regenerating islet-derived 3 gamma	4.61
10502801	H28	Histocompatibility 28	3.3
10364542	CFD	Complement factor D	3.09
10563602	Saa4	Serum amyloid A 4	2.81
10576757	Fcer2a	Fc receptor, IgE, low affinity II, alpha polypeptide	2.41
10466172	Ms4a1	Membrane-spanning 4-domains, subfamily A, member 1	2.39
10562169	Hamp	Hepcidin antimicrobial peptide	2.38
10551025	Cd79a	CD79A antigen	2.11
10531987	Gbp4	Guanylate binding protein 4	2.11
10437224	Mx2	Myxovirus (influenza virus) resistance 2	2.1
10441233	Mx1	Interferon-induced GTP-binding protein Mx1	1.96
10500677	CD2	CD2 antigen	1.85
10450675	H2-T24	Histocompatibility 2, T region locus 24	1.81
10451287	Isg15	ISG15 ubiquitin-like modifier	1.81
10531994	Mpa2l	Guanylate binding protein 6	1.79
10398121	Bdkrb1	Bradykinin receptor, beta 1	1.76
10399710	Rsad2	Radical S-adenosyl methionine domain containing 2	1.75
10435982	Btla	B and T lymphocyte associated	1.74
10468898	Lax1	Lymphocyte transmembrane adaptor 1	1.71
10547894	CD4	CD4 antigen	1.7
10444821	H2-Q8	Histocompatibility 2, Q region locus 8	1.69
10444236	H2-DMb2	Histocompatibility 2, class II, locus Mb2	1.67
10372652	Lyz1	Lysozyme 1	1.66
10574098	Nlrc5	NOD-like receptor C5	1.66
10601385	Tlr13	Toll-like receptor 13	−1.66
10560242	C5ar1	Complement component 5a receptor 1	−1.72
10541614	Clec4d	C-type lectin domain family 4, member d	−1.78
10347335	Slc11a1	Solute carrier family 11, member 1	−1.85
10416837	Irg1	Immunoresponsive gene 1	−2.08
10493831	S100a8	S100 calcium binding protein A8	−2.63
10349648	Ctse	Cathepsin E	−2.77
Cell adhesion molecule			
10433172	Glycam1	Glycosylation dependent cell adhesion molecule 1	1.98
10500677	CD2	CD2 antigen	1.85
10450675	H2-T24	Histocompatibility 2, T region locus 24	1.81
10547894	CD4	CD4 antigen	1.7
10444821	H2-Q8	Histocompatibility 2, Q region locus 8	1.69
10444236	H2-DMb2	Histocompatibility 2, class II, locus Mb2	1.67
10562720	Siglece	Sialic acid binding Ig-like lectin E	−1.78
10557862	Itgam	Integrin alpha M	−2.04
Transcription			
10404389	Irf4	Interferon regulatory factor 4 (TF)	2.06
10585276	Pou2af1	POU class 2 associating factor 1	2.09
10562812	Spib	Spi-B transcription factor (Spi-1/PU.1 related) (TF)	1.83
10360406	Ifi205	interferon activated gene 205	1.65
10390691	Nr1d1	Nuclear receptor subfamily 1, group D, member 1 (TF)	1.7
10460585	Fosl1	Fos-like antigen 1	−1.85

We then analyzed genes that are uniquely expressed in either *Fra-1*^*+/+*^ (see Additional file 1: Table S1) or *Fra-1*^∆/∆^ mice (see Additional file 2: Table S2) after bleomycin treatment. The *Fra-1*^∆/∆^ mice showed an up-regulation of chemokine (C-C motif) ligand 19 (*Ccl19,* 2.07-fold), macrophage-derived chemokine (*Ccl22,* 2.42-fold), chemokine (C motif) ligand 1 (*Xcl1,* 2.15-fold), chemokine (C-X-C motif) ligand 11 (*Cxcl11,* 2.53-fold), chemokine (C-C motif) receptor 4 (*Ccr4,* 1.75-fold), chemokine (C-C motif) receptor 8 (*Ccr8,* 2.13-fold) and chemokine (C-C motif) receptor 9 (*Ccr9,* 2.63-fold) (see Additional file 2: Table S2), whereas *Fra-1*^*+/+*^ mice showed an up-regulation of chemokine (C-X-C motif) receptor 2 (*Cxcr2,* 2.83-fold), interleukin 1 family, member 9 (*Il1f9,* 2.43-fold), chemokine (C-X-C motif) ligand 2 (*Cxcl2,* 3.78-fold), colony stimulating factor 2 receptor, alpha, low-affinity (*Csf2ra,* 2.02-fold) and interleukin 1 receptor-like 2 (*Il1rl2,* 1.85-fold) (see Additional file 1: Table S1). Of particular interest is the *Ccl22* chemokine, which has been recognized as a Th2 chemokine, and its involvement in the pathophysiology of pulmonary fibrosis has been documented. Belperio et al. have demonstrated that *Ccl22* and its receptor, *Ccr4,* are overexpressed in a mouse model of belomycin-induced fibrosis [26]. *Ccl22* and *Ccr4* levels are also increased in patients with IPF, and their expression has been detected on epithelium and macrophages, respectively [27]. Neutralization of *Ccl22* and *Ccr4* has been shown to lead to a significant reduction in lung inflammation during bleomycin-induced fibrosis [26,28]. Interestingly, our results here showed that the expression of *Ccl22* and *Ccr4* was significantly increased (2.42- and 1.75-fold, respectively) in bleomycin-treated *Fra-1*^∆/∆^ mice when compared to *Fra-1*^*+/+*^ mice.

Next, we analyzed genes that are uniquely down-regulated in bleomycin-treated *Fra-1*^∆/∆^ mice (see Additional file 2: Table S2), which included chemokine (C-C motif) receptor-like 2 (*Ccrl2,* -1.75-fold), bone morphogenetic protein 2 (*Bmp2,* -1.78-fold), and bone morphogenetic protein 3 (*Bmp3,* -1.78-fold). Similarly, *Fra-1*^*+/+*^ also showed uniquely down-regulated genes, including chemokine (C-X-C motif) receptor 5 (*Cxcr5,* -2.38-fold), chemokine (C-C motif) ligand 21A (*Ccl21a,* -1.72-fold), chemokine (C-C motif) ligand 27a (*Ccl27a,* -1.75-fold) and ciliary neurotrophic factor receptor (*Cntfr, -*1.75-fold) (see Additional file 1: Table S1). Taken together, the results of the present study have revealed that genetic disruption of Fra-1 differentially regulates a number of cytokines and chemokines in response to bleomycin, indicating a potential role for Fra-1 in cytokine and chemokine signaling during bleomycin-induced acute lung injury.

### Genes encoding proteins that are involved in the inflammatory response

*In Fra-1*^∆/∆^ mice treated with vehicle, we found up-regulation of some genes involved in the inflammatory response (Table 1) when compared to their *Fra-1*^*+/+*^ counterparts. These genes included regenerating islet-derived 3 gamma (*Reg3g,* 7.00-fold), cathepsin E (*ctse,* 3.61-fold), serine peptidase inhibitor, clade A, member 1B (*Serpina1b,* 4.61-fold) and serum amyloid A3 (*saa3,* 1.82-fold). On the other hand, the lack of Fra-1 led to the down-regulation of the expression of a few genes, including serine peptidase inhibitor, clade A, member 3 M (*Serpina3m, -*2.32-fold) and NLR family, CARD domain containing 5 (*Nlrc5, -*1.78-fold). Matrix metalloproteases (MMPs) play key roles in tissue repair and remodelling; but recent studies indicate a prominent role for the lysosomal proteinases, such as cathepsins, in the extracellular remodelling [29,30]. BAL fluid and extracellular space contain secreted cathepsins and their activities are controlled by endogenous inhibitors of cathepsins [31,32]. Likewise, serine peptidases are implicated in various biological processes such as wound healing and they also contribute to the development of pulmonary fibrosis and acute lung injury [33,34]. Endogenous serine peptidase inhibitors regulate the activities of serine peptidases. Imbalance in the activities of proteinases/peptidases and endogenous proteinases/peptidases inhibitors may contribute to deregulated protein degradation and resulting in the initiation of lung injury [35]. Serum amyloid A is an acute phase protein, induced by several inflammatory mediators and its serum level is elevated in various conditions like COPD [36], bronchial carcinoma [37], and cardiovascular disease [38]. Thus, it is likely that Fra-1 distinctly regulates proteinases/peptidases and their inhibitor’s expression to maintain lung homeostasis.

When we compared the gene expression patterns of *Fra-1*^∆/∆^ mice and their *Fra-1*^*+/+*^ littermates following bleomycin treatment, it was evident that there were many more up-regulated genes than down-regulated genes involved in inflammation in the lungs of *Fra-1*^∆/∆^ mice when compared to their *Fra-1*^*+/+*^counterparts (Table 2). We identified differential expression of genes that belong to the immunoglobulin family, specifically immunoglobulin joining chain (*Igj,* 17.25-fold) and immunoglobulin heavy constant mu (*Igh-6*, 7.43-fold) in *Fra-1*^∆/∆^ mice treated with bleomycin (Table 2). It has previously been shown that immunoglobulin concentrations are increased in immune disorders, such as rheumatoid arthritis [39], inflammatory bowel disease [40], and some respiratory disorders including asthma [41], cystic fibrosis [42], and idiopathic pulmonary fibrosis [43]. The up-regulation of immunoglobulin genes was also accompanied by an up-regulation of several genes involved in antigen presentation and antigen binding. This was true for some of the major histocompatibility genes, including histocompatibility 28 (*H28,* 3.3-fold), histocompatibility 2, class II, locus Mb2 (*H2-DMb2,* 1.67-fold), histocompatibility 2, T region locus 24 (*H2-T24,* 1.81-fold), and histocompatibility 2, Q region locus 8 (*H2-Q8,* 1.69-fold) (Table 2). Complement components such as complement factor D (*CFD,* 3.09-fold), and antigens such as CD79A antigen (*Cd79a,* 2.11-fold), CD2 antigen (*CD2,* 1.85-fold), and CD4 antigen (*CD4,* 1.70-fold) were differentially up-regulated in *Fra-1*^∆/∆^ mice (Table 2). The expression of other inflammatory genes, including regenerating islet-derived 3 gamma (*Reg3g,* 4.61-fold) and serum amyloid A 4 (*Saa4,* 2.81-fold), was also differentially up-regulated in *Fra-1*^∆/∆^ mice treated with bleomycin (Table 2). The *Reg3g* and Saa4 genes have now been consistently associated with pulmonary fibrosis and chronic inflammation [36,44]. On the other hand, we also noticed down-regulation of some genes in *Fra-1*^∆/∆^ mice, such as solute carrier family 11, member 1 (*Slc11a1, -*1.85-fold), S100 calcium binding protein A8 (*S100a8, -*2.63-fold), and cathepsin E (*Ctse, -*2.77-fold) (Table 2), when compared to *Fra-1*^*+/+*^ mice. Again, we analyzed genes that are uniquely expressed in either *Fra-1*^∆/∆^ (see Additional file 2: Table S2) or *Fra-1*^*+/+*^ (see Additional file 1: Table S1) mice in response to bleomycin. The results showed that major histocompatibility molecules such as histocompatibility 2, Q region locus 6 (*H2-Q6,* 2.35-fold), MHC class I like protein GS10 (*H2-gs10,* 2.12-fold), and complement components such as complement component 4B (*C4b,* 1.78-fold) were uniquely up-regulated in *Fra-1*^∆/∆^ mice treated with bleomycin (see Additional file 2: Table S2). In contrast, we found down-regulation of immunoglobulin heavy constant mu (*Igh-6,* -8.33-fold), histocompatibility 2, O region beta locus (*H2-Ob,* -3.12-fold), Cd226 antigen (*Cd226,* -1.75-fold), and complement receptor 2 (*Cr2,* -2.22-fold) in *Fra-1*^*+/+*^ mice after bleomycin treatment. Our previous study demonstrated that *Fra-1*^∆/∆^ mice showed increased levels of inflammation after bleomycin treatment [12]. Thus, our present data suggest that deregulation of the expression of immune response genes in *Fra-1*^∆/∆^ mice is the likely cause of the increased lung inflammation in *Fra-1*^∆/∆^ mice.

### Genes that encode extracellular matrix and cell adhesion molecules

The unique gene expression pattern in *Fra-1*^*+/+*^ mice (see Additional file 1: Table S1) treated with bleomycin suggested an increase in the expression of genes that encode extracellular matrix, such as collagen, type IV, alpha 1 (*Col4a1,* 1.96-fold), collagen, type IV, alpha 2 (*Col4a2,* 1.76-fold), collagen, type VI, alpha 1 (*Col6a1,* 1.70-fold), collagen, type VI, alpha 2 (*Col6a2,* 1.85-fold), collagen, type VI, alpha 3 (*Col6a3,* 1.98-fold), collagen, type XV, alpha 1 (*Col15a1,* 1.79-fold), a disintegrin-like and metallopeptidase (reprolysin type) with thrombospondin type 1 motif, 1 (*Adamts1,* 1.94-fold), and a disintegrin-like and metallopeptidase (reprolysin type) with thrombospondin type 1 motif, 2 (*Adamts2,* 2.07-fold). In contrast, *Fra-1*^∆/∆^ mice showed increased expression levels of laminin, alpha 1 (*Lama1,* 1.75-fold).

Among the various collagens, types I and III collagens are the most widely distributed in both airways and parenchymal structures [45]. To maintain normal structural properties of lung, the controlled distribution of these proteins is vital and their inappropriate accumulation in fibrotic lungs has been reported [46,47]. In contrast, the physiologic functions and abnormal deposition patterns of other collagens in the lung fibrosis are poorly understood. Our recent study showed an increased expression of the TGF-β1 and type-1-collagen genes in response to bleomycin at the end of 14 and 31 days of bleomycin treatment and demonstrated the presence of increased fibrosis in *Fra-1*^∆/∆^ mice [12]. However, we did not observe any differences in the expression levels of type I collagen or type III collagen, nor did we observe altered TGF-β1 gene expression in either genotype at the end of 5 days bleomycin treatment. It has been reported that excessive synthesis and deposition of ECM proteins is a general tissue response to an unresolved chronic inflammation [48]. Hence, we speculate that the persistence of increased inflammation in *Fra-1*^∆/∆^ mice is driven by the loss of Fra-1, while higher levels of fibrotic gene expression in the fibrotic stage (14 days) may contribute to the excessive deposition of ECM and disease severity seen in *Fra-1*^∆/∆^ mice. This point needs to be addressed in order to better understand the mechanisms underlying the increased fibrosis in *Fra-1*^∆/∆^ mice.

We found that a few genes known to be involved in general cell adhesion are affected by bleomycin treatment in *Fra-1*^∆/∆^ mice (Table 2). Glycosylation-dependent cell adhesion molecule 1 (*Glycam1,* 1.98-fold), cd2 antigen (*Cd2,* 1.85-fold), sialic acid binding Ig-like lectin E (*Siglece, -*1.78-fold), and integrin alpha M (*Itgam,* -2.04-fold) were differentially expressed in *Fra-1*^∆/∆^ mice treated with bleomycin when compared to *Fra-1*^*+/+*^ mice. In general, the expression of intercellular adhesion molecules is increased by inflammatory signals, which facilitates lymphocytes for higher adhesion and permeation into inflamed tissues. It has been shown that the glycam1 molecule is strongly expressed in inflammatory processes in order to modulate leukocyte trafficking [49]. Sialic acids present on the surface of all mammalian cells and play important roles in physiological and pathological processes, and their expression has been reported to decrease during immune cell activation [50]. Consistent with these findings, we also noted an up-regulation in the expression of *glycam1* and a loss of *siglece* in *Fra-1*^∆/∆^ mice when compared to *Fra-1*^*+/+*^*mice*.

We then analyzed the genes that are uniquely expressed in *Fra-1*^∆/∆^ (see Additional file 2: Table S2) or *Fra-1*^*+/+*^ mice (see Additional file 1: Table S1). *Fra-1*^∆/∆^ mice showed unique expression of genes that included major histocompatibility molecules such as histocompatibility 2, Q region locus 6 (*H2-Q6,* 2.35-fold), histocompatibility 2, T region locus 22 (*H2-T22,* 1.81-fold), histocompatibility 2, K1 region (*H2-K1,* 1.7-fold), laminin alpha 1 (*Lama 1,* 1.75-fold), and heparanase (*Hpse,* 1.98-fold). *Fra-1*^*+/+*^ mice showed unique expression of mesothelin (*Msln,* 2.16-fold), neuron-glia-CAM-related cell adhesion molecule (*Nrcam,* 2.1-fold), and a disintegrin and metallopeptidase domain 12 (*Adam 12,* 2.1-fold). Heparanase is an endoglucuronidase that cleaves heparan sulfate (HS) chains, resulting in HS fragments of 10 to 20 sugar units [51]. Overexpression of heparanase has been reported in numerous tumors, where it regulates angiogenesis and metastasis [52]. Furthermore, the ADAM gene family is associated with proteolytic, cell-cell, and cell-matrix interaction-promoting activities. Several investigations have shown a functional role for ADAMS in collagen deposition in cells and in lungs in which the ADAM gene was knocked down, thus revealing the functional dysregulation of this gene family in the lung fibrosis [53-55]. Thus, an alteration in the expression of cell adhesion molecules may represent another potential mechanism by which more fibrosis can occur in *Fra-1*^∆/∆^ mice.

### Fra-1-regulated genes involved in programmed cell death

The process of programmed cell death is known to play a major role in maintaining many biological processes, and inappropriate apoptosis can lead to disease conditions, either because cells experience an inappropriately prolonged survival or they die prematurely [56,57]. Studies using genetic models have demonstrated both cooperative and antagonistic roles of AP-1 family of proteins in modulating cell death in response to a variety of pro-apoptotic stimuli. For example, *c*-*Jun*^*-/-*^ mouse embryonic fibroblasts (MEFs) and liver cells show increased levels of oxidative stress and apoptosis [58]. Likewise, c-Fos also participates in both pro- and anti-apoptotic activities. For example, *c-Fos*^-/-^ MEFs undergo apoptosis when cultured *in vitro* and also display an increased susceptibility to UV-induced cell death [59]. Overexpression of Fra-1 also inhibits proliferation, induces apoptosis, and reduces the tumorigenicity of c6 glioma cells [60]. Consistent with a role for Fra-1 in apoptosis, we recently found that mouse embryonic fibroblasts lacking Fra-1 show an increased resistance to oxidant-induced cell death [61]. Fra-1 appears to uniquely up-regulate some genes modulating apoptosis in *Fra-1*^*+/+*^ mice (see Additional file 1: Table S1), including paternally expressed 3 (*Peg3,* 2.55-fold), the tumor necrosis factor receptor superfamily, member 10b (*Tnfrsf10b,* 2.03-fold), AXL receptor tyrosine kinase (*Axl,* 1.73-fold), Eph receptor A2 (*Epha2,* 1.7-fold), zinc finger matrin type 3 (*Zmat 3,* 1.76-fold), solute carrier family 40 (iron-regulated transporter, member 1) (*Slc40a1,* 1.70-fold), and EGL nine homolog 3 (*Egln3,* 1.81-fold). Similarly, *Fra-1*^∆/∆^ mice showed (see Additional file 2: Table S2) up-regulation of glutamate-cysteine ligase, catalytic subunit (*GCLC,* 1.70-fold) and down-regulation of lectin, galactose binding, soluble 12 (*Lgals 12,* -1.78-fold), Eph receptor A7 (*Epha 7,* -1.72-fold), and arachidonate 12-lipoxygenase (*Alox12, -*1.69-fold). It has been reported that kaempferol exerts an anti-oxidative and anti-apoptotic effects in HEI-OC1 cells treated with cisplatin through enhancing GCLC expression [62]. Consistent with this observation, we found that *GCLC* was induced in *Fra-1*^∆/∆^ mice. Our gene expression results from *Fra-1*^∆/∆^ mice are therefore in good agreement with the observation that inappropriate apoptosis can lead to exaggerated lung fibrosis.

### Validation of microarray data

Amongst several genes that were significantly affected by bleomycin, we randomly selected 17 genes according to the microarray results to confirm their differential expression by qRT-PCR. We confirmed that bleomycin treatment significantly induced the expression of *Il1a*, *Irf4*, *Reg3g,* and *Ccr4* and reduced the expression of *S100a8* in *Fra-1*^∆/∆^ mice when compared to similarly treated *Fra-1*^*+/+*^ mice (Table 3). These results confirmed the expression patterns of the microarrays. Next, we analyzed the genes that were uniquely expressed in both genotypes. The results revealed that *Fbln2* expression was significantly higher in *Fra-1*^*+/+*^ mice treated with bleomycin than in the vehicle-treated control group (Table 4); however, there was no difference observed in *Fra-1*^∆/∆^ mice. Similarly, *Fra-1*^∆/∆^ mice treated with bleomycin also showed significantly increased expression of *Hpse*, *Gclc*, *Runx3, Xcl1,* and *Cxcl11* when compared to the vehicle-treated group, but no differences were observed with their wild-type counterparts. These results further confirmed the expression patterns of the microarrays. According to the qRT-PCR results, there was a tendency for increased expression of *Ccr8* and *Ccl22* in the *Fra-1*^∆/∆^ mice treated with bleomycin as compared to the vehicle-treated mice, but the differences were not significant. Similarly, wild-type mice treated with bleomycin showed greater expression of *Col4a1* and *Tnfrsf10b* than did the vehicle controls. While these qRT-PCR results agree in general direction with the trends measured by microarray, the results did not reach statistical significance. Comparison of microarray and qRT-PCR results revealed discordance in 2 of the 17 genes selected. In the case of *Marco*, microarray analysis revealed no change in *Marco* expression in *Fra-1*^*+/+*^ mice but a significant increase in bleomycin-treated (3.08 ± 0.11) *Fra-1*^*+/+*^ mice when compared to vehicle-treated mice, as assayed by qRT-PCR. In the second case, the microarray results showed no change in the expression of *Snai2* in *Fra-1*^*+/+*^ mice, but the real-time RT-PCR results indicated a significant decrease (0.37± 0.15) in expression in these mice. The discordance in these two genes may be explained by the lower sensitivity of the microarrays.

**Table 3:** **Validation of differentially expressed genes induced by bleomycin in lung tissues from both *Fra-1***^***+/+ ***^**and *Fra-1***^**∆/∆ **^**mice**

**Affymetrix ID**	**Symbol**	**Gene title**	**Micro array**	**qRT-PCR**
			**KOT vs. WTT**	**KOT vs. WTT**
			**Fold change**	**Fold change**
10487588	Il1a	Interleukin 1 alpha	1.87	3.40 ± 1.68*
10404389	Irf4	Interferon regulatory factor 4 (TF)	2.06	3.29 ± 1.08*
10597420	Ccr4	C-C chemokine receptor type 4	1.78	1.68 ± 0.18*
10545569	Reg3g	Regenerating islet-derived 3 gamma	4.61	6.89 ± 0.86*
10493831	S100a8	S100 calcium binding protein A8	−2.63	-3.57±1.70*

Data are presented as mean ± SD (n=3-4). *P<0.05, WTT vs KOT after bleomycin treatment.

**Table 4:** **Validation of uniquely expressed genes induced by bleomycin in lung tissues from both *Fra-1***^***+/+ ***^**and *Fra-1***^**∆/∆ **^**mice**

**Affymetrix ID**	**Symbol**	**Gene title**	**Micro array**		**qRT-PCR**	
			**WTT vs. WTC**	**KOT vs. KOC**	**WTT vs. WTC**	**KOT vs. KOC**
			**Fold change**	**Fold change**	**Fold change**	**Fold change**
10540085	Fbln2	Fibulin 2	2.28	ND	2.35 ± 0.27*	ND
10531737	Hpse	Heparanase	ND	1.98	ND	1.57 ± 0.10*
10587266	Gclc	Glutamate-cysteine ligase, catalytic subunit	ND	1.70	ND	1.58 ± 0.27*
10509030	Runx3	Run related transcription factor 3	ND	2.02	ND	1.30 ± 0.14*
10359697	Xcl1	Chemokine (C motif) ligand 1	ND	2.15	ND	2.57 ± 0.48*
10531420	Cxcl11	Chemokine (C-X-C motif) ligand 11	ND	2.53	ND	4.72 ± 2.33*
10590242	Ccr8	Chemokine (C-C motif) receptor 8	ND	2.13	ND	1.58 ± 0.00^NS^
10574213	Ccl22	Macrophage-derived chemokine	ND	2.42	ND	1.73 ± 0.54^NS^
10576973	Col4a1	Collagen, type IV, alpha 1	1.96	ND	1.83 ± 1.08^NS^	ND
10416230	Tnfrsf10b	Tumor necrosis factor receptor superfamily, member 10b	2.03	ND	2.30 ± 1.09^NS^	ND
10357261	Marco	Macrophage receptor with collagenous structure	ND	2.03	3.08 ± 0.11*^†^	2.23 ± 0.21*
10433776	Snai2	Snail homolog 2 (Drosophila)	ND	-1.92	-2.23*^†^	-3.50 ± 1.95*

Data are presented as mean ± SD (n=3-4). *P<0.05, PBS vs bleomycin of same genotype. ^NS^ means non significant. ^†^ Result did not agree with microarray result.

### Analysis of selected microarray genes at different time points after bleomycin treatment

**Figure 1:**
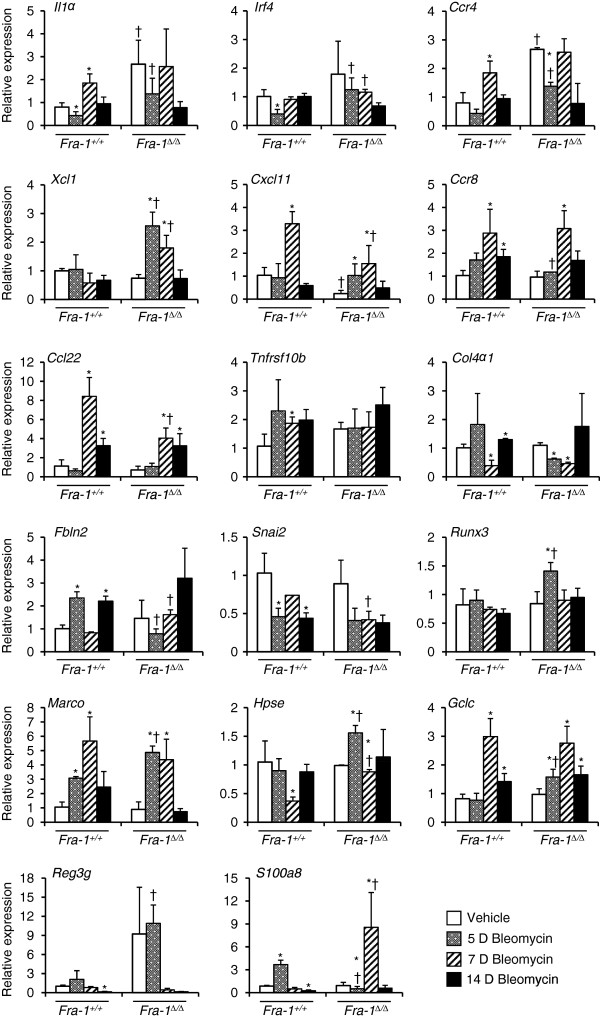
**Validation of the differences in mRNA expression between *Fra-1***^***+/+***^**and *Fra-1***^***Δ/Δ ***^**mice for selected microarray genes at different time points after bleomycin treatment.** Lung mRNA abundance was determined by quantitative real-time RT-PCR. The graphs represent the fold change over vehicle treated *Fra-1*^*+/+*^ controls after normalization with the expression of GAPDH. Results are mean ± SD for 3-4 mice in each group. ^*^p<0.05, PBS vs bleomycin; ^†^p<0.05, *Fra-1*^*Δ/Δ*^ vs *Fra-1*^*+/+*^ mice.

**Figure 2:**
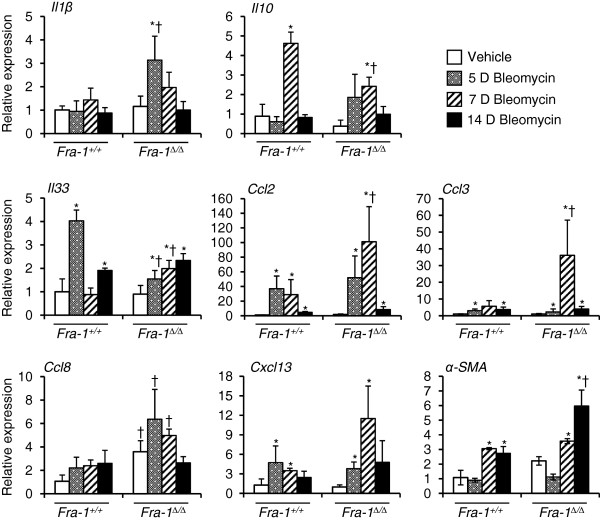
**Analysis of some gene expression between *Fra-1***^***+/+***^**and *Fra-1***^***Δ/Δ ***^**mice at different time points after bleomycin treatment.** Lung mRNA abundance was determined by quantitative real-time RT-PCR. The graphs represent the fold change over vehicle treated *Fra-1*^*+/+*^ controls after normalization with the expression of GAPDH. Results are mean ± SD for 3-4 mice in each group. ^*^p<0.05, PBS vs bleomycin; ^†^p<0.05, *Fra-1*^*Δ/Δ*^ vs *Fra-1*^*+/+*^ mice.

To identify the time course of gene induction by bleomycin, we analyzed samples at different time points for selected genes. All the genes that were used for microarray validation were also used for our analysis of temporal patterns of gene expression (Figure 1). The results revealed that most of the genes continued to show no significant differences between *Fra-1*^*+/+*^ and *Fra-1*^∆/∆^ at 7 and 14 days after bleomycin treatment. However, some of the genes were predominantly up-regulated (*Xcl1* and *S100a8*) or down-regulated (*Cxcl11* and *Ccl22*) in *Fra-1*^∆/∆^ mice only at 7 days following bleomycin treatment, when compared to the vehicle-treated control and *Fra-1*^*+/+*^ mice. We were further interested in the delayed response of some of the genes involved in lung fibrosis that did not exhibit significant differences in microarray results at 5 days (Figure 2). The results revealed a significant difference in the induction of C-C chemokines such as *Ccl2*, *Ccl3,* and *Ccl8* between *Fra-1*^*+/+*^ and *Fra-1*^∆/∆^ mice: *Fra-1*^∆/∆^ mice showed significantly elevated expression at 7 days following bleomycin treatment when compared to wild-type mice. Interestingly, excessive production of *Ccl2*, *Ccl3,* and *Ccl8* has been shown to aid the development of pulmonary fibrosis [63,64]. In addition to the up-regulation of *Ccl2*, *Ccl3*, and *Ccl8*, we observed a marked down-regulation of *Il10* and *Il33* in bleomycin-treated *Fra-1*^∆/∆^ mice. Previous studies have reported that *in vivo* IL-10 gene delivery before [65] and after bleomycin [66] administration suppresses the development of pulmonary fibrosis. Finally, fibroblast transdifferentiation has been shown to contribute to the pathology of pulmonary fibrosis. Therefore, we analyzed the expression of α-SMA, a marker for myofibroblasts. The results revealed that *Fra-1*^∆/∆^ mice treated with bleomycin had a significantly higher expression of α-SMA than did vehicle-treated control or *Fra-1*^*+/+*^ mice at 14 days. This result further supports the enhanced susceptibility of Fra-1-null mice to bleomycin-induced lung fibrosis.

## Conclusion

The factors that contribute to the pathogenesis of pulmonary fibrosis include persistent inflammation, generation of pro-inflammatory, pro-fibrotic and angiogenic mediators, alveolar epithelial cell injury, fibroblast differentiation, and poor apoptotic activity of the myofibroblasts. These deregulated cellular processes eventually lead to excessive deposition of extracellular collagen and pathological fibrosis [48,67,68]. The present mRNA expression profiling analysis has revealed an important role for Fra-1 in regulating components of complex regulatory networks controlling the lung injury and fibrosis. We found that *Fra-1*^∆/∆^ mice displayed some of the factors that contribute to pulmonary fibrosis, such as increased expression of pro-inflammatory genes and decreased expression of genes involving in apoptotic process during bleomycin treatment. Thus, we propose that strategies enhancing Fra-1 functions may represent a promising approach to mitigate pulmonary fibrosis.

## Methods

### Mice

Conventional deletion of *Fra-1* is embryonic lethal due to extra-embryonic tissue defects [69]. The mice bearing *Fra-1* “floxed” allele [69] (hereafter referred as Fra-1^FF^ mice) were obtained from Erwin F. Wagner (Spanish National Cancer Research Centre, Madrid, Spain). These mice are maintained on a mixed (C57BL6/129) background. Meox2 (Sox2)-Cre transgenic mice (C57BL6/129), in which Cre expression specifically restricted in embryo but not in extra-embryonic tissues, were obtained from the Jackson Labs. Meox2 Cre mice were crossed to Fra-1^F/F^ mice [70], in order to obtain *Fra-1*^*F/F*^-Meox2-Cre mice as described earlier [12]. Fra-1F/F mice with and without Cre are hereafter referred to as *Fra-1*^∆/∆^ and *Fra-1*^*+/+*^ genotypes, respectively.

### Bleomycin treatment

Bleomycin (0.075U) (APP Pharmaceuticals, LLC, Schaumburg, IL, USA) diluted in 30 μL of PBS was intratracheally administered to mice (n=3) (10-14 weeks old) as described previously [71]. Mice treated with PBS (n=3) served as controls. All experiments were conducted under a protocol approved by the institutional animal care use committee of the University of Illinois at Chicago. At the end of 5 days treatment, the left lungs were frozen immediately in RNAlater (Ambion) for subsequent microarray and qRT-PCR analysis.

### RNA isolation and labeling

Total RNA was isolated from *Fra-1*^*+/+*^ and *Fra-1*^∆/∆^ mice administered with PBS and bleomycin using Qiagen RNeasy micro kit (Cat no. 74004). RNA concentration and purity was determined before gene expression profiling using the Affymetrix MoGene 1.0ST v1 Array (gene array) (Affymetrix, Inc., Santa Clara, California). The microarray labeling, hybridization and processing was performed at the University of Illinois Research Resource Center according to the manufacturer’s protocol. Microarray data have been deposited in the National Center for Biotechnology Information Gene Expression Omnibus database (Accession number: GSE43695).

### Microarray data analysis

The raw probe signal intensities were quantile normalized over all samples, summarized with the robust multi-array average (RMA) algorithm [72] and log2 transformed with a median polish, using the Affymetrix Power Tools. We considered a transcript cluster (gene-level) to be reliably expressed in a sample if the Affymetrix implemented DABG (detection above ground) p-value was less than 0.05. We used local-pooled-error (LPE) estimates and robust statistical tests [73] for evaluating significance of each gene’s differential expression in a comparison (e.g., wild-type vs. wild-type with bleomycin treatment). The LPE estimation was shown to be powerful and effective in case of a small number of replicate arrays [73]. False discovery rate (FDR) was controlled at 1% using the LPE library for the R Statistical Package [74].

### Pathway analysis

We searched for any enriched pathways and biological processes among the differential genes in each comparison relative to the genes covered on the gene expression profiling platform using the NIH/DAVID (The Database for Annotation, Visualization and Integrated Discovery) [75]. Particularly, the following databases were interrogated: KEGG (Kyoto Encyclopedia of Genes and Genomes) [76] and GO (Gene Ontology) [77]. A minimum of 5 genes and the Benjamini corrected p-value less than 0.01 were used to call significantly enriched pathways or biological processes. There are distinct temporal phases during bleomycin-induced lung injury and fibrosis. To dissect the differential gene expression during bleomycin-induced initial lung injury, we have analyzed the gene expression profiles in *Fra-1*^*+/+*^ and *Fra-1*^∆/∆^ mice given PBS or bleomycin. We then compared the gene expression profiles in various categories: (1) differentially expressed genes in the lung tissue of *Fra-1*^∆/∆^ mice vs. *Fra-1*^*+/+*^ mice (Table 1), (2) differentially expressed genes induced by bleomycin in the lung tissue of *Fra-1*^*+/+*^ vs. *Fra-1*^∆/∆^ (Table 2), (3) unique gene expression induced by bleomycin in the lung tissue of *Fra-1*^*+/+*^ mice (see Additional file 1: Table S1), and (4) unique gene expression induced by bleomycin in the lung tissue of *Fra-1*^∆/∆^ mice (see Additional file 2: Table S2). The resulting gene lists were divided into several categories based on functional analysis in order to dissect Fra-1-dependent and -independent transcriptional programs.

### Validation of microarray analysis

Total RNA (1 μg) was reverse transcribed using qScript cDNA super mix (Quanta Biosciences, Inc. Cat no. 95048-100). qRT-PCR was performed using fluorogenic SYBR Green and detection system (Applied Biosystems). PCR was performed using primers listed in (Additional file 3: Table S3). For *Gclc*, *Marco* and *α-SMA*, TaqMan gene expression assays were purchased from Applied Biosystems (Foster City, CA). The cycle threshold (CT) values for each gene were normalized to that of GAPDH, and the relative value for PBS treated *Fra-1*^*+/+*^ was set as one arbitrary unit (AU). Values are shown as mean ± SD, with n=3-4 for each experimental condition. Student’s *T* test was used and p ≤ 0.05 was considered significant.

## Competing interests

The author’s declare that they have no competing interests.

## Authors’ contributions

SR and SPR were involved in the conception, delineation of hypotheses, and design of the study, as well as the analysis and interpretation of data. SR performed the experiments. NMR and WZ participated in bioinformatics analysis. SR and SPR wrote the manuscript. All the authors read and approved the final manuscript.

## Supplementary Material

Additional file 1: Table S1 Several unique genes expression induced by bleomycin in lung tissues of *Fra-1*^*+/+*^ mice. Click here for file

Additional file 2: Table S2 Several unique genes expression induced by bleomycin in lung tissues of *Fra-1*^∆/∆^ mice. Click here for file

Additional file 3: Table S3 Sequences of sybr green mouse primers used for qRT-PCR. Click here for file
